# Risk and protective factors for the mental health of displaced Ukrainian families in the Netherlands: study protocol of a 4-year longitudinal study

**DOI:** 10.1136/bmjopen-2024-089849

**Published:** 2025-04-02

**Authors:** Marjolein Missler, Ira Karaban, Ksenia Cheliuskina, Iryna Frankova, Natascha Dobrova-Krol, Marit Sijbrandij, M Olff, Maartje Schoorl, M L Duckers, Trudy Mooren

**Affiliations:** 1 Utrecht University Faculty of Social Sciences, Utrecht, The Netherlands; 2 ARQ Centrum’45, Diemen, The Netherlands; 3 Vrije Universiteit Amsterdam, Amsterdam, The Netherlands; 4 ARQ National Psychotrauma Centre, Diemen, The Netherlands; 5 Amsterdam Universitair Medische Centra, Amsterdam, The Netherlands; 6 LUBEC Treatment Center, Leiden University, Leiden, The Netherlands; 7 IMPACT, Diemen, The Netherlands; 8 Rijksuniversiteit Groningen, Groningen, The Netherlands; 9 Department of Clinical Psychology, Utrecht University, Utrecht, The Netherlands

**Keywords:** MENTAL HEALTH, Parents, Child, Family, Stress, Psychological, PUBLIC HEALTH

## Abstract

**Abstract:**

**Background:**

Over 6 million people have fled their homes in response to the full-scale invasion of Russian armed forces into Ukraine and are forcibly displaced since the start on 4 February 2022. Refugees, both adults and children, have a high risk of developing mental health disorders, in particular post-traumatic stress disorder, depression and anxiety disorders. Research into the mental health of Ukrainian families and their needs is urgently needed. The primary aim of this study is to increase our understanding of the consequences of potentially traumatic events for the mental health of parents and children. This may inform the development of mental health and psychosocial support interventions which can be readily implemented in the family context.

**Methods and analysis:**

We will conduct a four-wave longitudinal online survey study among Ukrainian families displaced to the Netherlands. This study is a part of the Nadiya data collection, intended to assess the mental health responses of Ukrainian refugee families to the stress of war, forced migration, family separation and adaptation to new circumstances in their hosting country. Participants are assessed at four time points, approximately 6 months apart. Data collection for T1 started in May 2023. We aim to recruit a total of n=1500 participants at T1, of which n=1000 adults (18 years and older) and n=500 children (8–11 years) and teenagers (12–17 years). To investigate symptom profiles and associated risk and protective factors among parents and children, we will use latent class growth modelling.

**Ethics and dissemination:**

The data collection procedure has been approved by the Ethical Committee of the Faculty of Social Sciences of Utrecht University. Data will be deposited, stored and shared using Utrecht University’s institutional research data repository Yoda. This research project is part of the Global Collaboration on Traumatic Stress; all authors are affiliated with this network. The findings will be published in peer-reviewed, open access journals and further disseminated through conference presentations, news updates at the project website and on the websites of the Dutch Society for Traumatic Stress Studies (www.ntvp.nl), and the European Society of Traumatic Stress Studies, as well as through media contributions.

**Trial registration number:**

The current study was registered on 26 March 2024 on The Open Science Framework (OSF): https://doi.org/10.17605/OSF.IO/9FP7U.

**STRENGTHS AND LIMITATIONS OF THIS STUDY**

- This 4-year longitudinal study includes a sample of both parents and children, drawn from community as well as clinical populations.
- Using latent class analysis and multilevel growth mixture modelling, we can identify different symptom trajectories as well as predictors of these trajectories.
- We measure a broad range of mental health indicators, accounting for expected heterogeneity in responses.
- The inclusion of sufficient adolescents and children might be a challenge, as well as sample attrition during the three follow-up measures.
- Another limitation of this study is the reliance on self-report questionnaires.

## Introduction

 Over 6 million Ukrainians have fled their homes in response to the full-scale invasion of Ukraine by Russian armed forces and are forcibly displaced since the escalation on 24 February 2022.^1^ While most forcibly displaced people (FDP) from Ukraine reside in neighbouring countries, more than 108 000 FDP reside in the Netherlands as of 24 February 2022.^2^ As the majority of the male citizens are requested to remain in Ukraine to defend the country, these FDP are mainly women and children living separately from their male relatives elsewhere. Notably, the enduring conflict jeopardises the mental health of a large proportion of Ukraine citizens, including those who are forcibly displaced or involved in the military. Research into the mental health of Ukrainian FDP families and their needs is therefore urgently needed.

Refugees and migrants have a high risk of developing symptoms of mental health disorders, in particular post-traumatic stress disorder (PTSD), depression and anxiety disorders.^3 4^ Negative mental healthcare consequences are not only at stake in adults,^5^ but also in children.^6^ In general, parental distress has been related to both externalising and internalising problems in children’s development.^7 8^ This holds especially true for refugee parents struggling with symptoms of traumatic distress^9^ or prolonged grief disorder.^10^ A potential mechanism behind this association is that distressed parents are less able to recognise and respond to the needs of their children^11^; see also Alisic *et al*.^12^ Importantly, children need their parents to be available for warmth, safety and support to ensure healthy development.^13^ For refugee parents who are struggling with (traumatic) distress and/or prolonged grief, it might be even more difficult to recognise their child’s signals, respond adequately, and express adequate levels of affection.^9 14^

Indeed, cross-sectional research in Germany showed high self-reported prevalence rates of anxiety, depression and general stress among Ukrainian FDP,^15^ which form a considerable threat to their quality of life.^15^ While being exposed to war-related violence and being forced to flee to another country threatens mental health in and of itself, symptoms of distress can be exacerbated by experiencing post-migration stressors, such as separation from family, loss of important social identities, financial problems, problems with employment or being underemployed and discrimination.^16^ These stressors have been consistently associated with poorer mental health in refugees,^17^,^19^ whereas access to healthcare may be restricted. While the initial response in the majority of European societies has been welcoming and demonstrated hospitality, most likely, many Ukrainian FDP are nevertheless exposed to these post-migration stressors. Moreover, for Ukrainian families, the continuation of the war in their home country—including the separation from (male) relatives who are forced to risk their lives—may serve as a significant stressor,^15^ overshadowing the protecting effect of the warm welcome they received in different European societies.

Previous research on mental health disorders in FDP generally tended to examine the prevalence, severity and predictors of various mental health disorders separately.^20^,^22^ This could overlook the heterogeneity of mental health responses (ie, the clustering of symptoms from different disorders within individuals)^23 24^ and the comorbidity of mental health disorders among FDP.^25^ Recently, patterns of post-migration stressors have been identified,^17^ showing subgroups of FDP with high, moderate and low post-migration problems. Moreover, two distinct classes were identified based on people primarily reporting fear for immigration (eg, not being allowed to stay in the host country, issues with refugee status) and people primarily struggling with the degree of social disconnection (feeling isolated, lonely and/or bored). The latter class tended to report more different potentially traumatic events compared with the fear for immigration class. However, these classes were linked to specific disorders (PTSD, depression) and not to (clusters of) mental health symptoms. Therefore, in this study, we intend to assess the combined mental health burden (including symptoms of a range of mental disorders) among adult and child Ukrainian FDP. More specifically, we will investigate which distinct profiles including symptoms of PTSD, anxiety, depression and prolonged grief disorder can be identified among displaced Ukrainian adults and children/teenagers. Additionally, we will examine predictive factors of the identified symptom trajectories. This will allow insight into the mental health status of Ukrainian displaced people of all ages and into the factors that are associated with various levels of mental health burden.

For adults, we hypothesise that a higher lifetime exposure to potentially traumatic events and a higher exposure to current post-migration stressors will be predictive of a symptom profile associated with higher symptom severity;^26 27^ see also Bogic *et al.*^28^ Furthermore, we hypothesise that the absence of the death of a loved one (ie, absence of grief),^29^ higher levels of resilience^30^ and higher perceived social support^31^ will be predictive of a symptom profile associated with lower symptom severity. For children/teenagers, we also hypothesise that a higher lifetime exposure to potentially traumatic events will be predictive of membership to a symptom profile associated with higher symptom severity. Furthermore, we hypothesise that the absence of the death of a loved one (ie, absence of grief), higher levels of well-being and general health will be predictive of membership to a symptom profile associated with lower symptom severity.^32^

To investigate symptom profiles and associated predictors thereof, we will use Latent Class Analysis (LCA).^33^ There are studies using LCA to detect symptom profiles including various mental health disorders among FDP. Symptom profiles of PTSD, depression, anxiety and somatic issues in a sample of treatment-seeking refugees have been examined.^26^ They identified three distinct symptom profiles: (1) highly severe symptoms, (2) severe symptoms and (3) moderate symptoms. Almost half of the participants were observed in either the high severe symptoms class or the severe symptoms class, with about 10% of participants in the moderate symptoms class. However, this was a cross-sectional study, targeting treatment-seeking refugees. Furthermore, Lenferink *et al*^27^ examined the symptom profiles of PTSD and depression within a general, non-treatment-seeking, refugee sample of different origins. They identified four symptom profiles: (1) no symptoms, (2) low PTSD/moderate depression, (3) moderate PTSD/depression and (4) high symptoms. The majority of the participants were observed in the no-symptom class, whereas the minority of participants were observed in the high-symptom class. Importantly, they used dichotomous scores (disorder is present or not) to describe the different symptom profiles. In contrast to the previously mentioned studies, in the study by Ryu *et al*,^34^ the majority of the sample could be classified as the ‘high trauma with high comorbidity’ group. However, these authors focused only on symptom clusters of trauma, social withdrawal, aggression and depression among Korean adolescents who fled from northern Korea.

The current study will build on these earlier findings by (1) employing a longitudinal design, enabling us to detect changes in FDP’s mental health over time; (2) including symptoms of four of the most common mental disorders among refugees, enabling us to identify heterogeneous symptom profiles; and (3) targeting a community sample of adults and children. To the best of the authors’ knowledge, symptom profiles of the combination of symptoms of PTSD, depression, anxiety and grief have not yet been explored, not within a refugee sample, nor with adults or children/teenagers in general. In regard to both adults and children, we expect to identify classes of various symptom severity combinations of PTSD, depression, anxiety and grief. Based on previous research,^26 27^ we hypothesise that the majority of our non-treatment-seeking sample of Ukrainian FDP will be observed in a no symptom/low symptom severity profile and the minority in the high symptom severity profile. Notably, in the aforementioned studies, adult FDP were studied. Research on the combined mental health burden among FDP children is currently lacking. Hence, there is a particular pressing need to study mental health responses among young refugees.^35^

In short, this unforeseen humanitarian crisis confronts host countries with new and extra challenges with regard to protecting the mental health of Ukrainian families. Currently, their mental health is not systematically monitored, and it is thus unclear what kind of support these families might need. This jeopardises not only the health of Ukrainian adults, but poses also a major risk to their children, who are vulnerable to developing mental health problems themselves. Therefore, the primary aim of this study is to increase our understanding of (1) the consequences of potentially traumatic events for the mental health needs of families and (2) possibilities to alleviate these (eg, risk and supporting factors). Our second aim is to inform the development of supportive interventions for refugee families, which can be readily implemented. While our study is focused on the mental health of Ukrainian refugees residing in the Netherlands, it holds the potential to add to and improve knowledge on the experienced mental health burden of refugee groups from different backgrounds. This can contribute to the development of culturally appropriate intervention strategies to address mental health problems among refugee parents and might aid in the prevention of subsequent problems among their children.

## Method

### Study design

We will conduct a four-wave longitudinal online survey study. This study is part of the Nadiya (the Ukrainian word for ‘hope’) research project, intended to assess the mental health responses of Ukrainian forcibly displaced families to the stress of war, forced migration, family separation and adaptation to new circumstances in their hosting country (in this case The Netherlands). Nadiya is a Dutch research collaboration between Utrecht University, VU University (Vrije Universiteit) Amsterdam, and the universities of Amsterdam and Leiden and ARQ Psychotrauma Expert Group. Participants will be assessed at four time points (ie, T1, T2, T3, T4), approximately 6 months apart. Data collection started in May 2023 (T1); October 2023 (T2); June 2024 (T3); and November 2024 (T4), respectively. The current study was registered on 26 March 2024 on The Open Science Framework (OSF): https://doi.org/10.17605/OSF.IO/9FP7U.

### Participants

#### Eligibility

The current study focuses on the Ukrainian population forcibly displaced to the Netherlands as a consequence of the full-scale invasion of Ukraine by Russian armed forces on 24 February 2022. All participants must be at least 8 years old to participate and arrived in the Netherlands after 24 February 2022. Participants who resided in the Netherlands prior to 24 February 2022 are excluded.

#### Sample size

We aim to recruit a total of n=1500 participants at T1, of which n=1000 adults and n=500 children (8–11 years) and teenagers (12–17 years). The desired sample size was decided in collaboration with the Dutch public health services based on earlier population-based prospective studies^36 37^ taking into account an expected attrition rate of 50–60% at each wave. This sample size will be sufficient to perform analysis of longitudinal models (eg, latent growth modelling) and estimate the weight of risk and protective factors of mental health over the four time points.

### Recruitment and procedure

#### Recruitment

Participants are recruited through various channels, namely: (1) social media platforms (eg, Facebook, Instagram, Telegram), (2) collaborating partners (eg, Dutch public health services, Opora foundation), (3) the social network of the authors and mental healthcare clinics and universities whom the authors are affiliated with and (4) face-to-face contact of the authors with potential participants during visitation of known large-scale residences of the Ukrainian population. This means that the final sample will be drawn from both clinical and non-clinical populations. However, to reduce the risk of ascertainment bias, we will compare the characteristics of the sample with Statistics Netherlands data on the Ukrainian population in the Netherlands,^38^ as well as with prior research among Ukrainians forcibly displaced to the Netherlands.^39^ Recruitment material consists of digital and printed flyers and is available in Ukrainian, Russian and English. We also used digital banners available in Ukrainian. The participant information brochure and informed consent are adapted to the legal requirements of the age of the participant.

#### Procedure

Nadiya is focused on families with children, and therefore, we aim to recruit as many members of the same family as possible. Participants belong to one of three groups based on their age: (1) adults, aged 18 or older, (2) teenagers, between the age of 12 and 17 and (3) children, between the age of 8 and 11. All participants will provide written informed consent before entering the study. For children aged 8–11 years, parents provide consent; for youngsters (12–15 years), we will collect informed consent from both the child as well as the parent. For children aged 16 and 17, only the child will be asked to provide consent. In accordance with the Declaration of Helsinki, it will be clearly indicated on the informed consent form that study participation is entirely voluntary and that participants can withdraw from participation at any time without negative consequences for them or their child. After providing consent, each participant is asked to complete an online Qualtrics survey appropriate to their age. All questionnaires are designed to be as brief as possible to decrease participation burden. The adult, teenage and children survey takes respectively about 30, 20 and 15 min to complete. All who participated during T1 will be invited via email to participate in the subsequent follow-up assessments (T2–T4). All participants who completed the survey on all four time points will partake in a gift card lottery, of which 10% of the participants will receive a gift card worth €15.

### Data management

The collected data will be (pseudo-anonymised) and hosted at Utrecht University (Yoda). The data collection procedure has been approved by the Ethical Committee of Faculty of Social Sciences of Utrecht University (FETC, 22–313).

### Measures

For an overview of all measures, please refer to online supplemental table A1.

#### Adult survey

##### Primary outcome: parental distress symptomatology

*Depressive symptoms* will be measured with the Patient Health Questionnaire (PHQ-9).^40^ This scale consists of 9 items and measures the presence and frequency of depressive symptoms during the past 2 weeks. Respondents can indicate how often they experienced each symptom on a scale from 0 (Not at all) to 3 (Nearly every day). An example item is: ‘Little interest or pleasure in doing things’. Total scores range from 9 to 27 and can be computed by summing the individual items. Kroenke *et al*^39 40^ reported excellent psychometric properties, with Cronbach’s alpha varying from 0.86 to 0.89.

*Anxiety symptoms* will be measured by means of the General Anxiety Disorder (GAD-7) questionnaire.^41^ This questionnaire consists of 7 items and measures the presence and frequency of anxiety symptoms during the past 2 weeks. Respondents can indicate how often they experienced each symptom on a scale from 0 (Not at all) to 3 (Nearly every day). An example item is: ‘not being able to stop or control worrying’. Total scores range from 7 to 21 and can be computed by summing the individual items. Previous research reported high internal consistency of the GAD-7, with a Cronbach’s alpha of 0.89 across different samples in the general population.^42 43^

*Post-traumatic stress symptoms* will be measured by the abbreviated 8-item PTSD Checklist for the Diagnostic and Statistical Manual of Mental Disorders (DSM)-5 (PCL-5).^44 45^ This scale measures the presence and burden of PTSD symptoms during the past month, such as intrusive thoughts and avoidance of thought and emotions. An example item is: *‘*Feeling very upset when something reminded you of the stressful experience?’. Items can be answered on a 4-point scale ranging from 0 (Not at all) to 5 (Extremely). Total item scores range from 8 to 40 and can be computed by summing the individual items. In their review, Forkus *et al*^46^ indicated that the abbreviated 8-item version of the PCL-5 has excellent internal consistency (Cronbach’s alpha of 0.93).

*Prolonged grief*, as measured by the abbreviated 16-item Traumatic Grief Inventory Self-Report (TGI-SR).^47 48^ This version of the TGI-SR measures symptoms corresponding with the former DSM-IV ‘persistent complex bereavement disorder’ diagnosis (Prolonged Grief Disorder in DSM-IV-TR). An example item is: ‘I had intrusive thoughts and images associated with his/her death’. Participants can rate the extent to which they experience each symptom on a 5-point scale, ranging from 1 (Never) to 5 (Always). Total scores can range from 16 to 90 and can be computed by summing the individual items. The 16-item version of the TGI-SR has high internal consistency, with a Cronbach’s alpha of 0.95.^47^ Participants only complete this scale when they report having lost a close family member or friend.

##### Secondary outcomes

*Family-related information*, namely number of people in the household, relationship status, number of (grand)children (including number of (grand)children below the age of 18), close family members that are currently living with the respondent, close family members currently living in Ukraine and close family members currently serving in the army.

*Lifetime stressful experiences* will be measured by the Life Events Checklist (LEC).^49^ This scale consists of 17 items. Participants were asked to indicate ‘yes’ on this scale when they experienced, witnessed or learnt about a specific event or ‘no’ if they were never exposed to the listed event. An example item is: serious accident, fire or explosion. Psychometric research showed good psychometric properties of the LEC, including high convergent validity with other measures of exposure to potentially traumatic events.^50^

*Post-migration stressors and daily hassles*, as measured by the Post-Migration Living Difficulties Checklist (PMLDC).^51 52^ This scale consists of 17 items. Participants will be asked to indicate to what degree the listed difficulties had been a problem for them since their arrival in The Netherlands. Items can be answered on a 5-point scale, varying from ‘Was not a problem/did not happen’ (0) to ‘A very serious problem’ (4). An example item is: ‘Communication difficulties’. Total scores can be computed by summing the individual items and range from 0 to 68. The PMLDC has been used in many studies with refugee populations.^53^,^55^

*General health* will be measured by the Symptoms and Perceptions (SaP) questionnaire,^56^ which consists of 28 items referring to different physical and psychological concerns. Example items are: fatigue/tiredness, nausea and headache. Participants can indicate whether or not they experienced a symptom, and if so, for how long and whether or not they consulted a general practitioner. In this study, we only asked participants to indicate whether or not they experienced the listed concerns during the past month. Total scores (ranging between 0 and 28) can be computed by summing the individual items, with higher scores referring to a higher level of symptomatology. The total scale showed good psychometric properties.^56^

*Quality of life* will be measured by the one-item Quality of Life Health Questionnaire (EQ-5D-5L).^57^ Participants can indicate their general health status on a Visual Analogue Scale from 0 (worst health imaginable) to 100 (best health imaginable). Feng *et al*^57^ reported excellent psychometric properties.

*Perceived social support* will be measured by the Social Support List.^58^ This scale consists of 12 items which can be answered on a 4-point scale, with response options varying from 1 (Never or rarely) to 4 (Very often). Total scores range from 12 to 48, with higher scores indicating a higher level of perceived social support. Next to the total score, subscale scores can be computed for the following dimensions: ‘Daily support’; ‘Support with problems’; and ‘Appreciation’. An example item is: ‘Does it ever happen that someone reassures you?’ (support with problems subscale). Kempen and Van Eijk^58^ reported adequate psychometric properties for the total scale as well as for the subscales.

*Resilience* will be measured by the Resilience Evaluation Scale.^59^ This scale consists of 9 items, which refer either to the underlying dimension self-confidence (items 1, 7, 9) or self-efficacy (items 2, 3, 4, 5, 6). An example item is: ‘I can easily adjust in a difficult situation’. Participants can indicate the extent to which they agree with each statement on a 5-point scale, ranging from 0 (completely disagree) to 4 (completely agree). Total scores range from 0 to 36 (total scale); 0 to 12 (self-confidence subscale) and 0 to 20 (self-efficacy subscale) and can be computed by summing the individual items. Van der Meer *et al*^59^ reported excellent psychometric properties: internal consistency for the total scale varied from 0.83 (Dutch version) to 0.90 (English version).

*Current serviceutilisation*, as measured by one item adopted from the Adjust study^60^: *Which services have you used in the past month when you were distressed?* Participants can indicate the services they have been using, for example, online support, coaching and/or psychotherapy.

*Parenting* will be measured by the War Child Parenting questionnaire^61^ (adapted for the current study). The scale consists of 24 items, which can be answered on a 6-point scale varying from 0 (Never) to 3 (Often) or 4 (I do not know) and 5 (I do not want to answer). Total scores range from 0 to 72 and can be computed by summing the 0–3 scores. Subscale scores can be computed for parental warmth and sensitivity (14 items) and harsh parenting (5 items). Participants only complete this questionnaire if they report to have at least one (grand)child under the age of 18. Miller *et al*^61^ reported high internal consistency of the total scale (Cronbach’s alpha 0.87) as well as for the responsiveness subscale (0.84). Internal consistency of the harsh parenting subscale was adequate (0.76). For the current study, the validity and meaning of the translated items (in Ukrainian and Russian)—which had been developed for use in Syrian war-stricken parents—was checked in a focus group of four Ukrainian mothers reflecting on the items one by one. We did not include the items on Parenting Knowledge as we set out to assess behaviour more than attitudes; and we left out one item which we deemed too difficult and therefore open for ambiguity: ‘When my child misbehaves, I am able to use consequence that match the severity of his/her misbehavior’. We changed ‘them’ into ‘him/her’ to also be able to refer to one child. Lastly, we changed the answering categories by adding a response option ‘never’, as was strongly recommended by the focus group participants.

*Parental evaluation of their child’s health* will be measured with 3 items from the Patient-Reported Outcomes Measurement Information System questionnaire.^62^ Parents are asked to rate their child’s physical and mental health as well as their child’s ability to reach developmental milestones on a 5-point scale, ranging from 1 (poor) to 5 (excellent). Furthermore, parents are asked whether a doctor has informed them that their child has specific difficulties, such as a developmental disorder (eg, autism spectrum disorder) or a chronic condition (eg, asthma).

### Children (8–11 years old)

#### Primary outcome: distress symptomatology

*Lifetime stressful experiences and current traumatic stress* will be measured by the Child and Adolescent Trauma Screen (CATS)^63^ questionnaire. This instrument is based on the DSM criteria for PTSD (intrusions, avoidance, negative alterations in cognitions and mood, and hyperarousal). The questionnaire asks children whether a potentially traumatic event (PTE) has happened to them or not. The 15 items focus on natural disasters, accidents, experiencing or seeing violence at home or in the community, sexual abuse, traumatic loss, medical procedures or war. If at least one PTE has happened to the child, there are an additional 20 items inquiring about symptoms of traumatic stress, with response options varying from 0 (Never) to 3 (Almost always). An example item here is: ‘Trouble falling or staying asleep*’*. Subsequently, 5 items measure whether the symptoms interfere with key areas of functioning (getting along with others, school/work, hobbies, family relationships and general happiness). Sachser *et al*^63^ reported good reliability and validity for both the original as well as translated versions.

#### Secondary outcomes

*Childsocial-emotional functioning* as measured by the Strengths and Difficulties Questionnaire (SDQ).^64^ This questionnaire will only be completed by children 10 years or older. The items refer to strong points as well as difficulties experienced by the child. The 25 items measure emotional symptoms, conduct problems, hyperactivity-inattention, peer problems and prosocial behaviour. Each item can be answered on a 3-point scale, with response options 0 (Not true), 1 (Somewhat true) and 2 (Certainly true). Total scores can be computed by summing the individual items. In this study, the internalising (emotional symptoms and peer problems) and externalising (conduct problems and hyperactivity) dimensions will be used.^8^ Previous research using the dimensions reported high internal consistencies.^65^

*Quality of life and general well-being* will be measured by the KIDSCREEN-27 Questionnaire.^66^ The KIDSCREEN-27 is a shortened version of the original KIDSCREEN-52^67^ and consists of 27 items across 5 dimensions: physical well-being (5 items); psychological well-being (7 items); parents and autonomy (7 items); social support and peers (4 items); and school environment (4 items). Items can be answered on a 5-point scale, with response options varying from ‘Never’ to ‘Always’ or from ‘Not at all’ to ‘Extremely’. Rasch scores can be computed for each dimension, which are then transformed into a distribution with a mean of 50 and a SD of 10. Higher scores are indicative of a better quality of life. The shortened version that will be used in this study showed high validity in psychometric research.^66^

#### Teenagers (12–15 years old)

The questionnaire for teenagers is identical to the child version (8–11 years old), also in terms of primary and secondary outcomes. There is, however, one additional questionnaire (in case the child has lost a close family member or friend):

*Prolonged grief* will be measured by the Traumatic Grief Inventory for Children.^68^ This scale consists of 16 items measuring symptoms of prolonged grief disorder. An example item is ‘In the past month, did you miss ___ very much?’ Response options vary from 0 (Never) to 4 (Always). Total scores can be computed by summing the individual items and range from 0 to 64. This self-reported version of this questionnaire is not yet validated for children 8–12 years.

#### Baseline characteristics

Demographic characteristics of participants will be collected, namely age, gender identity, educational level, income, employment situation, relationship status, type of housing in the Netherlands and importance of religion in daily life. Furthermore, we asked participants the date of their first arrival in the Netherlands, and the part of Ukraine they lived in before the Russian invasion.

#### Translations

All surveys are made available in Ukrainian, Russian and English. Participants can choose which language they would like to read. The formal Ukrainian or Russian version of a questionnaire was used whenever this was available (for an overview, see online supplemental table A1 in Appendix A). When a Ukrainian or Russian version of the instrument was not available, authors who are native speakers in Ukrainian and Russian collaborated in translating the instrument, following translation and back-translation procedures. The translation (Ukrainian and Russian) of the War Child questionnaire was informed by a focus group of n=3 adult Ukrainian mothers to further improve the validity of the instrument.

### Statistical analysis

A multilevel-based three-step LCA^34^ will be implemented to identify distinct subgroups (ie, symptom profiles) of people that share a similar pattern of mental health responses^69^ based on cross-sectional data (ie, data from the first wave). After finalising data collection from waves 1–4, we will apply multilevel growth mixture modelling.^70^ In case of missing data, we will perform Little’s Missing Completely At Random (MCAR) test. If the missingness is random, we will use the maximisation of expectation technique^70 71^ to impute missing data. If the rate of missingness is low, we will use baseline data to test for systematic differences between completers and non-completers.

Based on data from the first wave, we will estimate a Latent Class model and investigate the frequency of each identified class-solution thereof. In other words, we will estimate which symptom profiles can be identified and how many participants share the same symptom profile. Then, by estimating latent class growth models,^72^,^74^ we will be able to detect potential different trajectories of responses to forced migration over time (T1–T4).

For both analyses, after checking for multicollinearity, we will examine the relationship between explanatory variables (ie, potential predictors) and the previously estimated class-solution membership. In other words, the third step provides insight into which factors may predict membership to the previously identified symptom profiles. To test the predictive value of a variable, we will first conduct one-way analysis of variance and χ^2^ tests to investigate for which variables the class-solution groups may differ (eg, participants ascribed to a certain symptom profile). If class-solution groups differ significantly on a variable mean or distribution, this variable will be regarded as a potential predictor. Then, using all identified potential predictors, we will conduct a multinomial regression analysis with all the identified potential predictors to determine how well these predict each class-solution group. We will adjust the p value using the Bonferroni method to correct for multiple testing.

The outlined analyses will be applied to data from adults and children/teenagers separately.

For adults, we are interested in which class solutions (ie, symptom profiles) emerge from the following domains of mental health outcome responses: post-traumatic stress (as measured by the PCL-5); general anxiety (as measured by the GAD-7); depression (as measured by the PHQ-9); and grief (as measured by the TGI-SR). The predictive value of four domains of explanatory variables will be investigated:

1. Stressful experiences: number of potentially traumatic events during lifetime, death of a loved one since February 2022, region in Ukraine participants lived in prior to the invasion of the Russian armed forces, time of arrival in the Netherlands, family currently living in Ukraine and family currently active within the Ukrainian army.
2. Post-migration factors: current accommodation type in the Netherlands, current employment status in the Netherlands and current presence of post-migration stressors (type, total number, perceived burden).
3. Individual factors: age, gender, education level, current relationship status, current degree of religious belief, current general health, current service use, current quality of life, current degree of resilience and current perceived social support.
4. Parenting factors: number of children, number of children younger than 18 years of age, current parental functioning and current parental evaluation of child health.

In regard to children and teenagers, their cross-sectional data will be initially merged into one larger dataset. If the sample size allows, we will make a further distinction between data of children and teenagers and apply the intended statistical analysis to the datasets separately. We are interested in which class solutions (ie, symptom profiles) emerge from the following domains of mental health outcome responses: post-traumatic stress (as measured by the CATS, part 2 and 3); internalising and externalising mental health symptoms (as measured by SDQ); and grief (as measured by the TGI-SR). The predictive value of two domains of explanatory variables will be investigated:

1. Stressful experiences: number of potentially traumatic events during lifetime and death of a loved one since February 2022.
2. Individual factors: age, gender, education and general well-being regarding five domains (physical well-being, psychological well-being, parental relations and autonomy, social support and peers and school environment).

Furthermore, if the sample size allows, we want to estimate the predictive value of parent–child relationship quality on symptom profile (class-solution) membership. To do this, we will extract a subset of data from adults, specifically from the participants who report having children under the age of 18. Then, the following steps will take place:

1. From the subset data of parents, parental assessment of child–parent relationship quality will be included in the multinomial regression analysis as a potential predictor of children/teenagers class-solution membership (ie, in the exploration of relevant predictors of symptom profiles of children/teenagers).
2. The outlined analyses will be applied to the subset data of parents. When conducting the multinomial regression analyses, data of children/teenagers on child–parent relationship quality will be included as a potential predictor of parents’ class-solutions membership (ie, in the exploration of relevant predictors of symptom profiles of parents).

All statistical analysis will be performed using R .4.3.0.^75^

### Patient and public involvement

Representatives of the Ukrainian refugee community in the Netherlands were involved in the research project in several ways: (1) participation in focus groups to translate questionnaires not yet available in Ukrainian language; (2) pilot testing of the online questionnaire; (3) recruitment. We visited Ukrainian residences and discussed how to best approach and recruit participants with Ukrainian families. Also, we asked them to inform others about the project. Results of this project will be disseminated through our website (news articles) and short movie clips targeted at Ukrainian children and adolescents. Also, we will invite participants and members of the Ukrainian community to several meet-ups, during which we explain our main findings (levels of distress in the Ukrainian community, predictors of distress) and will ask for input as to how to translate these findings to supportive interventions and policies.

## Ethics and dissemination

The data collection procedure has been approved by the Ethical Committee of the Faculty of Social Sciences of Utrecht University (FETC, 22-313). All researchers involved in this study are active in the Global Collaboration on Traumatic Stress; the Nadiya project is listed on its website to further promote international collaboration. The research findings will be published in peer-reviewed, open access journal articles. Participants will be kept up to date about the progress of the study through regular newsletters. Dissemination of the findings among researchers, clinicians and policymakers will further take place through conference presentations, and via news updates at the project website and on the websites of the Dutch Society for Traumatic Stress Studies (www.ntvp.nl) and the European Society of Traumatic Stress Studies, as well as through (written) media contributions. With consent from the participants, data will be deposited, stored and shared using Yoda, which is Utrecht University’s institutional research data repository. It is registered as such with re3data.org. Yoda complies with Utrecht University’s Information Security policy for data classified as public, internal use or sensitive.

## supplementary material

10.1136/bmjopen-2024-089849 online supplemental appendix 1
