# Supplementary material for: Risk and protective factors for the mental health of displaced Ukrainian families in the Netherlands: study protocol of a 4-year longitudinal study
Source: BMJ Open. 2025 Apr 2;15(4):e089849. doi: 10.1136/bmjopen-2024-089849 (PMC11966996; doi:10.1136/bmjopen-2024-089849)
Supplement: online supplemental appendix 1 [file bmjopen-15-4-s001.docx]

**APPENDIX A.**

**Table A1.**

*Overview of included instruments, and their translation information.*

| **Time-**  **point** | **Survey** | **Questionnaire** | **Measuring Domain** | **Ukrainian translation** | **Russian translation** |
| --- | --- | --- | --- | --- | --- |
| T1-T4 | C, T | Child and Adolescent Trauma Screen (CATS) **^1^** | Part 1: Stressful and potentially traumatic experiences;  Part 2 and 3: post-traumatic stress | FV | FV |
| T1-T4 | A, C, T | Demographics **^2^** | E.g. age, education, relationship status, current occupation, current housing, time of arrival in the Netherlands etc. | AV | AV |
| T1-T4 | A | EQ-5D-5L **^3^** | Quality of life | AV | AV |
| T1-T4 | A | General Anxiety Disorder 7 (GAD-7) **^4^** | Anxiety | FV | FV |
| T1-T4 | C, T | KIDSCREEN-27 **^5^** | Well-being and quality of life | AV | FV |
| T1-T4 | A | Life Events Checklist, adapted version for Forcibly Displaced People (LEC) **^6^** | Stressful and potentially traumatic experiences | AV | AV |
| T1-T4 | A | Which services have you used in the past month when you were distressed?**^7^** | Service use | AV | AV |
| T2-T4 | A | Frequency of alcohol and tabaco use**^2^** | Alcohol and Tabaco use | AV | AV |
| T1-T4 | A | Patient Health Questionnaire 9 (PHQ-9) **^8^** | Depression | FV | FV |
| T1-T4 | A | Post-Migration Living Difficulties (PMLD) **^9^** | Post-migration stress and daily hassles | AV | AV |
| T1-T4 | A | PTSD Checklist for DSM-5, abbreviated 8-item (PCL8-5) **^10^** | Post-traumatic stress | FV | FV |
| T1-T4 | A | PROMIS, selected items **^11^** | Parental evaluation of child health | AV | AV |
| T1-T4 | A | Resilience Evaluation Scale (RES) **^12^** | Resilience | AV | AV |
| T1-T4 | A | Social Support List (SSL-12) **^13^** | Perceived social support | AV | AV |
| T1-T4 | C, T | Strength and Difficulties Questionnaire (SDQ) **^14^** | General mental health | AV | AV |
| T1-T4 | A | Symptoms and Perceptions (SaP) **^15^** | General physical health | AV | AV |
| T1-T4 | A | Traumatic Grief Inventory Self-Report plus, abbreviated 16-item (TGI-SR+) **^16^** | Traumatic grief | FV | FV |
| T1-T4 | C, T | Traumatic Grief Inventory for Children (TGI-C) **^17^** | Traumatic grief | AV | AV |
| T1-T4 | A | WarChild Parenting **^18^** | Parental functioning | AV | AV |

**Note.** T1 – *First data collection wave*; T2 – *Second data collection wave*; T3 – *Third data collection wave*; T4 – *Fourth data collection wave*

***Note.*** AV – *Adult Survey (18yrs and older)*; T – *Teenage Survey (12-17 yrs)*; C – *Child Survey (8-11 yrs)*

***Note***. FV – *Formal version (existing standard translation)*; A – *Author version (translated by the authors)*

***Note****.* **^1^** Sachser et al. (2017); **^2^** Questions formulated by the authors; **^3^** Feng et al. (2021); **^4^** Spitzer et al. (2006); **^5^** *The Kidscreen Group Europe* (2006); **^6^** Weathers et al. (2013); **^7^** Questions adapted from the ADJUST COVID-19 study (Lotzin et al., 2022); **^8^** Kroenke et al. (1999); **^9^** Questionnaire developed and used in the STRENGTHS study (de Graaff et al., 2022); **^10^** Geier et al. (2020); **^11^** Dutch-Flemish PROMIS (n.d.); **^12^** Van der Meer et al. (2018); **^13^** Van Eijk et al. (1993); **^14^** Goodman (1997); **^15^** Yzermans et al. (2016); **^16^** Lenferink et al. (2022); **^17^** Dyregrov et al (2001); **^18^** Questionnaire used in study by Miller et al. (2020).
