# Supplementary material for: Risk and protective factors for the mental health of displaced Ukrainian families in the Netherlands: study protocol of a 4-year longitudinal study
Source: BMJ Open. 2025 Apr 2;15(4):e089849. doi: 10.1136/bmjopen-2024-089849 (PMC11966996; doi:10.1136/bmjopen-2024-089849)
Supplement: Supplementary file 2 [file bmjopen-2024-089849.reviewer_comments.pdf]

PEER REVIEW HISTORY

BMJ Open publishes all reviews undertaken for accepted manuscripts. Reviewers are asked to complete a checklist review form and are provided with free text boxes to elaborate on their assessment. These free text comments are reproduced below.

ARTICLE DETAILS

Title (Provisional)

Risk and Protective Factors for the Mental Health of Displaced Ukrainian Families in The Netherlands: Study Protocol of a Four-year Longitudinal Study

Authors

Missler, Marjolein; Karaban, Ira; Cheliuskina, Ksenia; Frankova, Iryna; Dobrova-Krol, Natascha; Sijbrandij, Marit; Olff, M; Schoorl, Maartje; Duckers, ML; Mooren, Trudy

---

VERSION 1 - REVIEW

**Reviewer**	**1**
**Name**	**Hodes, M.**
**Affiliation**	**Imperial College London, Division of Psychiatry**
**Date**	**26-Oct-2024**
**COI**	**nil**

The authors rightly state in their introduction that there has been a large influx of refuges from Ukraine into European countries and there is a dearth of evidence about their experiences of war, displacement and resettlement and impact on psychological distress. This protocol for a well-designed four-wave longitudinal online survey study amongst Ukrainian families displaced to the Netherlands will significantly push forward our knowledge in this area. The study will be valuable as it will include approximately 1000 adults, mainly women, and their offspring (approx. 500) so will provide data about family relationships as well as individual risk factors and the impact of ongoing stressors. The longitudinal design will enable associations and probable causal pathways to be unpacked. The protocol is well organised and generally clear. The study is already underway so any significant changes in protocol and design are now not feasible.

However there are some areas that need expanding:

1. Sampling

Further discussion is needed about sample recruitment because of risk of sample ascertainment bias. The sample comes from “Participants are recruited through various channels, namely: (1) social media platforms (eg. Facebook, Instagram, Telegram), (2)

collaborating partners (e.g. Dutch public health services, Opora foundation), (3) the social network of the authors and mental health care clinics and universities whom the authors are affiliated with, and (4) face-to-face contact of the authors with potential participants during visitation of known large-scale residences of the Ukrainian population".

This is a mixture then of clinical and community populations that include volunteers not a true population based sample

2. Sample attrition

The authors should specify how they'll address bias from sample attrition at follow-up and missing data.

3. The protocol should have a clearer section about strengths and limitations including address the biases described in 1 and 2 above and limitation of the measures and potentially small sample size especially of the children and adolescents.

4. Ethical considerations: there should be expansion in describing how consent will be obtained especially for children and adolescents' participation.

More minor amendments:

1. English needs improving : eg introduction. This sentence needs changing:

The enduring conflict includes all elements to fear mental health consequences in a large proportion of Ukraine citizens and those involved in the military, including those who are forcibly displaced.

2. Check References

This reference appears twice:

Byrow, Y., Liddell, B., O'Donnell, M., Mau, V., McMahon, T., Bryant, R., Benson, G., & Nickerson, A.(2022a). Profiles of post-migration stressors and mental health in refugees: A latent class analysis.Psychiatry Research, 311, 114494. https://doi.org/10.1016/j.psychres.2022.114494

VERSION 1 - AUTHOR RESPONSE

Reviewer: 1
Dr. M. Hodes, Imperial College London
Comments to the Author:
The authors rightly state in their introduction that there has been a large influx of refuges from Ukraine into European countries and there is a dearth of evidence about their experiences of war, displacement and resettlement and impact on psychological distress. This protocol for a well-designed four-wave longitudinal online survey study amongst Ukrainian families displaced to the Netherlands will significantly push forward our knowledge in this area. The study will be valuable as it will include approximately 1000 adults, mainly women, and their offspring (approx. 500) so will provide data

about family relationships as well as individual risk factors and the impact of ongoing stressors. The longitudinal design will enable associations and probable causal pathways to be unpacked. The protocol is well organised and generally clear. The study is already underway so any significant changes in protocol and design are now not feasible.

Thank you for your positive and thoughtful feedback on our study. Below, we respond to the points that you have raised one-by-one.

1. Sampling
Further discussion is needed about sample recruitment because of risk of sample ascertainment bias. The sample comes from "Participants are recruited through various channels, namely: (1) social media platforms (eg. Facebook, Instagram, Telegram), (2) collaborating partners (e.g. Dutch public health services, Opora foundation), (3) the social network of the authors and mental health care clinics and universities whom the authors are affiliated with, and (4) face-to-face contact of the authors with potential participants during visitation of known large-scale residences of the Ukrainian population".
This is a mixture then of clinical and community populations that include volunteers not a true population based sample

Indeed, given the difficulties with recruiting refugee populations (Djelantik et al., 2022) we will not be able to recruit a true population-based sample. We have added on p. 9, line 2: 'This means that the final sample will be drawn from both clinical and non-clinical populations.' However, we take several measures to mitigate the risk on ascertainment bias (p.9; line 3-5): 'we will compare the characteristics of the sample with Statistics Netherlands data (Statistics Netherlands, 2024) on the Ukrainian population in the Netherlands, as well as with prior research among Ukrainians forcibly displaced to the Netherlands (Research and Data Center, 2024).'

2. Sample attrition
The authors should specify how they'll address bias from sample attrition at follow-up and missing data.

We have added the following lines to the Statistical Analysis section (p.15; line 3-6): In case of missing data, we will perform Little's MCAR test. If the missingness is random, we will use the maximization of expectation technique (Liu and Brown, 2013) to impute missing data. If the rate of missingness is low, we will use baseline data to test for differences between completers and non-completers.

3. The protocol should have a clearer section about strengths and limitations including address the biases described in 1 and 2 above and limitation of the measures and potentially small sample size especially of the children and adolescents.

We have adapted our Strengths and Limitations section according to your suggestions (p.3; lines 4-12), including the risk for sample attrition during the study and potential difficulties with the inclusion of children and adolescents.

4. Ethical considerations: there should be expansion in describing how consent will be obtained especially for children and adolescents' participation.

We have added this information now on p. 9 (lines 13-19): 'All participants will provide written informed consent before entering the study. For children 8-11 years, parents provide consent; for youngsters (12-15 years), we will collect informed consent from both the child as well as the parent. For children aged 16 and 17, only the child will be asked to provide consent. In accordance with the Declaration of Helsinki, it will be clearly indicated on the informed consent form that study participation is entirely voluntary and that participants can withdraw from participation at any time without negative consequences for them or their child.'

More minor amendments:
1. English needs improving : eg introduction. This sentence needs changing:
The enduring conflict includes all elements to fear mental health consequences in a large proportion of Ukraine citizens and those involved in the military, including those who are forcibly displaced.

Thank you for pointing to this sentence, we have adapted it as follows (p.4; lines 9-11): 'Notably, the enduring conflict jeopardizes the mental health of a large proportion of Ukraine citizens, including those who are forcibly displaced or involved in the military.'

Furthermore, we have checked the entire manuscript on correct English language use. We have made adaptations on the following places (p.5, line 28; p.7, lines 7 and 13, lines 23-24, and line 28. We have updated the dates that data collection started for T3 and T4 on page 8 (lines 10-11). Some additional changes are made on:

- p. 9, lines 18 and 19
- p. 11, lines 3 and 5
- p. 12, lines 22 and 23
- p.13, line 19
- p.13, lines 29 and 30
- p.15, lines 12, 14, and 29
- p.17, lines 4 and 28

2. Check References
This reference appears twice:
Byrow, Y., Liddell, B., O'Donnell, M., Mau, V., McMahon, T., Bryant, R., Benson, G., & Nickerson, A.(2022a). Profiles of post-migration stressors and mental health in refugees: A latent class analysis.Psychiatry Research, 311, 114494.
https://doi.org/10.1016/j.psychres.2022.114494

Thank you for noting, we have deleted one of the duplicates (p.21) and, after checking all references, made some minor adaptations to the Reference list (p.19, line 13 and p.21, line 7).

References

Djelantik, A. A. A. M. J., Van Es, C. M., Lahuis, A. M., & Mooren, N. (2022). The challenges of conducting mental health research among resettled refugee populations: An ecological framework from a researchers' perspective. *International Journal of Mental Health*, 1–19. https://doi.org/10.1080/00207411.2022.2069962

Liu, Y., & Brown, S. D. (2013). Comparison of five iterative imputation methods for multivariate classification. *Chemometrics and Intelligent Laboratory Systems*, *120*, 106–115. https://doi.org/10.1016/j.chemolab.2012.11.010

Statistics Netherlands (2024). Oekraïners in Nederland [Ukrainians in The Netherlands]. Retrieved 26 January 2025, from https://longreads.cbs.nl/asielenintegratie-2024/oekrainers-in-nederland/.

Research and Data Center (2024). Tijdelijk thuis? De positie van Oekraïense vluchtelingen in Nederland [Temporarily at home? The position of Ukrainian refugees in the Netherlands]. Memorandum 2024-1. Den Haag: WODC.

VERSION 2 - REVIEW

**Reviewer**	**1**
**Name**	**Hodes, M.**
**Affiliation**	**Imperial College London, Division of Psychiatry**
**Date**	**07-Mar-2025**
**COI**	

Thank you for the revisions. I think all the points have been addressed now and the paper is ready for publication.
